# Mob Psychology

**Published:** 1910-10-22

**Authors:** 


### Mob Psychology.

The cases which occur of individuals who lose all control over themselves and are blown about by every capricious change of their passions, are sad enough, and often inexplicable. To the observant mind they present instances of the biblical "possession by devils." In fact, an individual in this helpless condition is virtually obsessed by the mob-spirit. Few, indeed, have tried adequately to explain these phenomena which are invariably associated with a great crowd. The individual is lost: he loses his identity, his self-control, and his judgment. In his veins courses some mysterious fluid which breaks down every barrier, and leaves the man as helpless as if he were under the influence of alcohol. For a crowd is not simply an aggregate of isolated individuals, a mere sum total of component parts. As in the chemical world, a compound possesses a different aspect and different properties from its ingredients, so a crowd has a personality of its own which is inseparably bound up with its component individuals, but at the same time is greater than they and different in expression. A crowd may be, in fact, a superhuman monster, whose caprices change from moment to moment.

Sometimes a man who finds himself in a crowd becomes a mere automaton, a being without judgment. With relaxed moral fibre comes that perversion of mind which results in the destruction of valuable property which he himself may have devoted years of his life to possess. The curious telepathic sympathy of his co-mates in destruction acts upon him like strychnine on the frog, and he becomes hypersensitive to stimulus. Floating as he does on the crest of a mighty wave of emotionalism, he forgets that he is being rapidly borne towards the rocky shore on which he must inevitably be dashed. Sometimes, indeed, we find in the individual this hopeless condition of unrestraint, and at such a time we do not regard the patient as capable of exercising a right judgment on anything. In fact, under some pretence or other, such patients are closely watched to prevent them doing damage either to themselves or other people.

The anomaly, therefore, of an election becomes all the more apparent when we reflect that it is to produce this unreliable condition of affairs, and to destroy individual judgment that the chief efforts of some speakers on both sides are directed. The bigger the crowd, in many cases the bigger the error, and where there is no man there only is there no error. But it is astonishing that we should look to pathological subjects for the settlement of questions which are momentous if ever there were momentous questions. We appeal to Philip drunk, and endeavour to keep him in such a condition that he will never again become sober. Even the Greeks, whose medical skill we are inclined to regard with so much pity, were less credulous than we. It was certainly more likely that utterances of a fume-besotted woman, in the hands of clever priests, would lead the Greeks to victory than that the demagogues of Athens would save the State. In politics there is danger in numbers, for in most cases a crowd is a *locus classicus* of morbid pathology. It is difficult at present to understand the origin of these peculiar phenomena as manifested in crowds. A man who attends a revivalist meeting will, at the suggestion of the preacher, wave a handkerchief to signify that he wishes to be saved, but, on leaving the meeting, he will realise his folly with disgust. To punish the members of a crowd is as foolish as to sentence a corporation of a town to a flogging. The offending personality is far too elusive. It is to be hoped, however, that before long, consideration of these evils will lead to a radical change in our electioneering system. Truth cannot be extracted from a well of frenzied prejudice—it always dies before help can reach it.

